# The Hypothalamic-Pituitary-Gonadal Axis in Men with Schizophrenia

**DOI:** 10.3390/ijms24076492

**Published:** 2023-03-30

**Authors:** Agnieszka Matuszewska, Krzysztof Kowalski, Paulina Jawień, Tomasz Tomkalski, Dagmara Gaweł-Dąbrowska, Anna Merwid-Ląd, Ewa Szeląg, Karolina Błaszczak, Benita Wiatrak, Maciej Danielewski, Janusz Piasny, Adam Szeląg

**Affiliations:** 1 Department of Pharmacology, Wroclaw Medical University, J. Mikulicza-Radeckiego 2, 50-345 Wroclaw, Poland; 2 Department of Biostructure and Animal Physiology, Wroclaw University of Environmental and Life Sciences, C.K. Norwida 25/27, 50-375 Wroclaw, Poland; 3 Department of Endocrinology, Diabetology and Internal Medicine, Tadeusz Marciniak Lower Silesia Specialist Hospital–Centre for Medical Emergency, A.E. Fieldorfa 2, 54-049 Wroclaw, Poland; 4 Department of Population Health, Division of Public Health, Wroclaw Medical University, Bujwida 44, 50-345 Wroclaw, Poland; 5 Department of Maxillofacial Orthopaedics and Orthodontics, Wroclaw Medical University, Krakowska 26, 50-425 Wroclaw, Poland

**Keywords:** schizophrenia, sex hormones, HPG axis, testosterone, hypogonadism, estradiol, prolactin, hyperprolactinemia, psychotropic drugs, male

## Abstract

Schizophrenia is a severe mental disorder with a chronic, progressive course. The etiology of this condition is linked to the interactions of multiple genes and environmental factors. The earlier age of onset of schizophrenia, the higher frequency of negative symptoms in the clinical presentation, and the poorer response to antipsychotic treatment in men compared to women suggests the involvement of sex hormones in these processes. This article aims to draw attention to the possible relationship between testosterone and some clinical features in male schizophrenic patients and discuss the complex nature of these phenomena based on data from the literature. PubMed, Web of Science, and Google Scholar databases were searched to select the papers without limiting the time of the publications. Hormone levels in the body are regulated by many organs and systems, and take place through the neuroendocrine, hormonal, neural, and metabolic pathways. Sex hormones play an important role in the development and function of the organism. Besides their impact on secondary sex characteristics, they influence brain development and function, mood, and cognition. In men with schizophrenia, altered testosterone levels were noted. In many cases, evidence from available single studies gave contradictory results. However, it seems that the testosterone level in men affected by schizophrenia may differ depending on the phase of the disease, types of clinical symptoms, and administered therapy. The etiology of testosterone level disturbances may be very complex. Besides the impact of the illness (schizophrenia), stress, and antipsychotic drug-induced hyperprolactinemia, testosterone levels may be influenced by, i.a., obesity, substances of abuse (e.g., ethanol), or liver damage.

## 1. Introduction

Schizophrenia is a chronic mental disorder that affects approximately 1% of people worldwide [1]. There are not any known biomarkers of schizophrenia, and the diagnosis is based on the observations of clinical symptoms [2]. For many years, it was thought that schizophrenia occurred with similar frequency in both sexes. However, more recent data indicate that schizophrenia affects men more often than women [3,4]. The clinical course of schizophrenia can be highly variable. Psychopathology distinguishes between positive symptoms, negative symptoms, and cognitive impairment. Positive symptoms are often referred to as psychotic or productive symptoms; these include delusions and hallucinations. Negative symptoms consist of blunted affect, decreased motivation, decreased energy, anhedonia, social withdrawal, and reduced spontaneous expressions [2,5]. Cognitive impairment refers to the impairment of memory and executive functions [6].

Medicine is increasingly paying attention to the different course of conditions by sex. Schizophrenia in men usually manifests earlier than in women. The peak incidence in men occurs between the ages of 18 and 25 years, while in women it is approximately 4 years later [7,8]. Furthermore, women experience a second peak in incidence during menopause [9,10]. In men, schizophrenia has a more severe course [11], presents with lower efficacy of antipsychotics [12], and the clinical presentation is more often dominated by negative symptoms [13]. The pathogenesis of these phenomena is not fully understood; however, physiological discrepancies in the functioning of men and women, including sex hormones, are likely to be important.

Testosterone is an important androgen with a role in many processes related to development, maturation, and ageing in men [14]. During puberty and beyond, it influences the occurrence of secondary sexual characteristics, causes morning erections, stimulates spermatogenesis, increases libido, and has anabolic effects [15]. Decreased testosterone levels in adult men lead to reproductive dysfunction, as well as fat gain, changes in lipid metabolism, reduced muscle mass and strength, decreased bone mineral density, predispose to anemia, lead to impaired memory and concentration, and a depressed mood [16,17]. High testosterone levels may be connected to the aggressive behaviour [18].

This review aims to present the current knowledge and analyse factors that may influence testosterone levels in men with schizophrenia. We consider the effects of the disease itself (schizophrenia), and antipsychotics on sex hormone levels and prolactin levels. This article also discusses other common problems in this group of patients that can disrupt the hypothalamic-pituitary-gonadal axis (HPG axis), such as stress, obesity, alcohol abuse, and liver damage.

## 2. Material and Methods

The cited articles were selected from *PubMed*, *Web of Science*, or *Google Scholar* in English or Polish. Different combinations of keywords were used: “schizophrenia”, “sex hormones”, “gender”, “male”, “men”, “reproductive system”, “testosterone”, “HPG axis”, “schizophrenia pharmacotherapy or treatment”, “antipsychotics drugs”, “hyperprolactinemia”, “obesity”, “liver disease”, and “alcohol or ethanol”. We did not limit the time or the type of the publications.

## 3. Neuroendocrinology—The Relationship between the Nervous System and the Endocrine System

**Figure 1:**
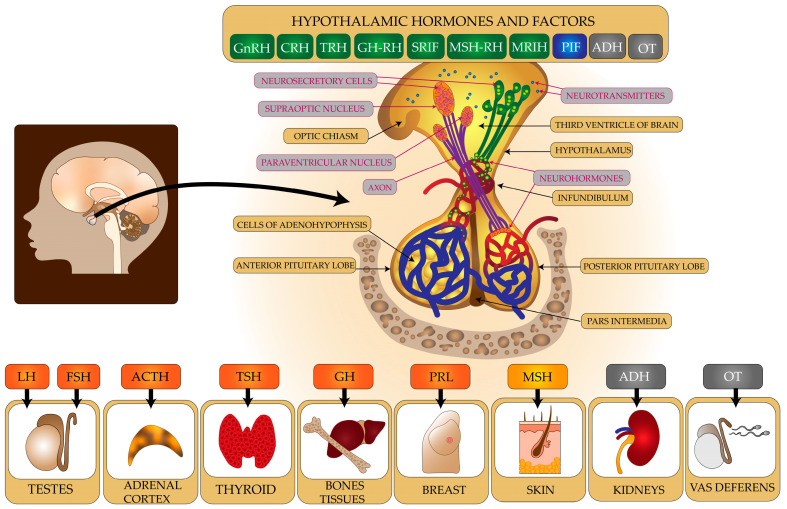
A diagram illustrating the relationships between the CNS and the endocrine system.

The nervous system and endocrine system are interconnected anatomically and functionally (Figure 1). The hypothalamus is crucial in this respect. It is a small neural structure (weighing less than 2–5 g in an adult) located in the central nervous system (CNS) in the lower part of the diencephalon. This structure is formed by the bottom of the third ventricle and the lower sections of its lateral walls [19].

The hypothalamus plays an overarching role in endocrine regulation by synthesizing and secreting numerous neurohormones [20]. Hypothalamic neurons produce and release into the pituitary portal circulation the following hormones: gonadoliberin (GnRH), corticoliberin (CRH), thyrotropin-releasing hormone (TRH), hormones that regulate growth hormone release (somatoliberin (GH-RH), somatostatin (SRIF)), hormones that regulate melanocyte-stimulating hormone (MSH-RH, MRIH), and prolactin-inhibiting factor (PIF) (dopamine). Hypothalamic neurons also have the ability to produce antidiuretic hormone (ADH) and oxytocin (OC). They are produced in the cell bodies of neurons within supraoptic and paraventricular nuclei and then transported along the axons to the posterior lobe of the pituitary gland where they are accumulated [21].

The hypothalamus connects to the pituitary gland by the infundibular stalk [19]. The pituitary gland is located inside the cranium within the sella turcica of the sphenoid bone. It consists of anterior (glandular), intermediate (pars intermedia), and posterior (neural) parts. The anterior part of the pituitary gland secretes into the blood luteinising hormone (LH) and follicle-stimulating hormone (FSH) to regulate gonadal function; adrenocorticotropic hormone (ACTH) to stimulate the adrenal cortex to produce cortisol; thyroid-stimulating hormone (TSH) to stimulate the thyroid gland to produce thyroxine and triiodothyronine; as well as growth hormone (GH) and prolactin (PRL). The pars intermedia of the pituitary gland produces melanocyte-stimulating hormone (MSH) [22]. The posterior lobe of the pituitary gland stores ADH and OC, which are produced by neurons in the hypothalamus [21]. Hormones released from the pituitary gland affect distant organs, tissues, and cells throughout the body.

The hypothalamus integrates information obtained from the endocrine and nervous systems. It has connections to many brain regions including the forebrain, limbic structures, and the brainstem. The hypothalamus also regulates the function of the autonomic nervous system (ANS) [23]. Hence, the production and secretion of neurohormones and factors by the hypothalamus may be influenced by signals received from other parts of the nervous system and the endocrine system [24].

## 4. Hypothalamic-Pituitary-Gonadal (HPG) Axis in Men

In a healthy adult male, approximately 5–10 mg of testosterone is produced per day—the vast majority of which (approximately 95%) is produced in the Leydig cells in the testes [25]. The remaining amount of testosterone is produced in the adrenal cortex and as a result of peripheral metabolism of precursors. Most of testosterone in the blood is bound to proteins. Sixty-six percent (66%) of testosterone is coupled to sex hormone-binding globulin (SHBG) and 30% of testosterone to albumin [25]. Free testosterone is biologically active and accounts for only 2–4%. It has the ability to interact with receptors on target cells.

**Figure 2:**
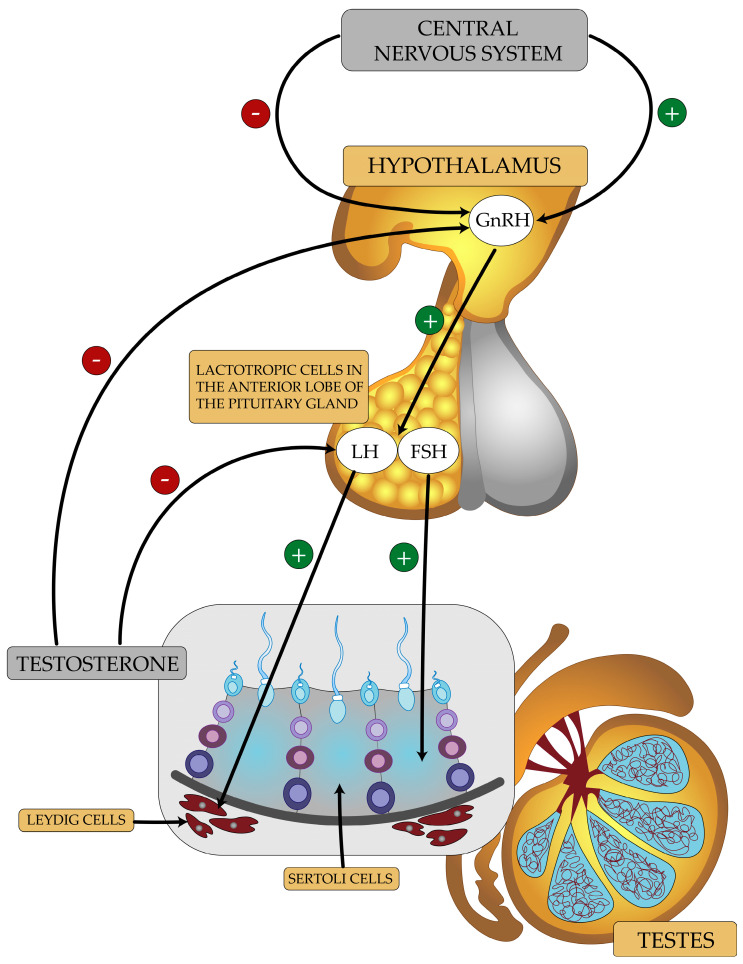
A diagram illustrating the basis for the functioning of the HPG axis in men.

The hypothalamic-pituitary (HP) axis is involved in the regulation of testosterone production in men (Figure 2). GnRH neurons located in the arcuate nucleus of the hypothalamus and preoptic region secrete gonadoliberin. GnRH released in a pulsatile manner binds to a membrane receptor on pituitary gonadotrophs [26]. This stimulates the cells of the anterior lobe of the pituitary gland to secrete gonadotropic hormones—LH and FSH [27]. LH binds to LHR receptors on the surface of Leydig cells and stimulates them to produce and secrete testosterone [15]. Testosterone diffuses into the seminiferous tubules and, together with its active metabolite, dihydrotestosterone (DHT), stimulates the maturation of reproductive cells [28]. FSH stimulates Sertoli cells to produce regulatory molecules, e.g., inhibin B, and nutrients needed to maintain spermatogenesis [29].

Numerous simple and complex neural, hormonal, and metabolic mechanisms are involved in the regulation of the HPG axis. GnRH release is regulated by the CNS and ANS [30,31]. For example, the neuropeptide kisspeptin has the ability to bind to KISS1R receptors on GnRH neurons. This stimulates the release of GnRH and gonadotropins [32,33]. The neurotransmitters, glutamate and noradrenaline, stimulate the HPG axis, whereas GABA inhibits it [27]. Hormonal regulation involves the direct action of hormones on the axis, causing stimulation or inhibition of the release of another hormone. An increase in serum testosterone levels causes, by feedback, a decrease in the secretion of GnRH, LH, and FSH. On the other hand, when the serum testosterone levels are too low, the release of GnRH, LH, and FSH is increased in a healthy man [34,35]. Testosterone can be converted to estradiol under the influence of aromatase in tissues. Estrogens inhibit the secretion of GnRH, LH, and FSH [36,37]. Sertoli cells produce inhibin. Inhibin B inhibits the release of FSH [38]. Hormone secretion is also influenced by direct metabolic substrates or products (metabolic regulation) [39].

## 5. Pathogenesis of Schizophrenia

Despite ongoing research, the etiology of schizophrenia is not well known. The pathogenesis of the disease is considered to be multifactorial, and both genetic and environmental factors are involved in its development [40]. Based on the studies on twins, it is known that schizophrenia exerts a high level of heritability, reaching 60–80% [2,41,42]. These findings were the impulse to search for genes responsible for the disease development. Recent, large-scale genomic studies confirmed a complex polygenic background of the schizophrenia [2]. In a genome-wide association study (GWAS), Trubetskoy et al. [43] found, in patients with schizophrenia and healthy controls, common variant associations at 287 distinct genomic loci in genes expressed in the neurons of the central nervous system (excitatory and inhibitory). Further analysis identified 120 genes (including 106 that codes proteins) that are probably connected to these *loci*. Fifteen products of these genes were recognized as synaptic proteins, both pre- and postsynaptic. These proteins are, among others, involved in endocytosis (*SNAP91*), signal transmission, and differentiation and organization of the synapses (*DLGAP2*, *LRRC4B*, *GPM6A*, *PAK6*, *PTPRD*). Many genes encode receptor proteins of the voltage-gated chloride (*CLCN3*) and calcium channels (*CACNA1C*), glutamate (*GRM1*) or GABA (*GABBR2*) metabotropic receptors, or the subunit of the ligand-gated NMDA receptor (*GRIN2A*). What is important is that gene expressions in which enrichment of common variant associations were found, was not restricted to any specific area of the brain and was present in excitatory and inhibitory neurons. This finding argues against the fact that brain dysfunction is limited to its particular areas and explains the diverse spectrum of symptoms of schizophrenia [43].

The association of SNPs with many genes, including genes coding the major histocompatibility complex (MHC), neuregulin 1 (*NRG1*), potassium channel (*KCNH2*), dysbindin, and many other proteins present in the brain was described [44]. Some other studies revealed common variation at genes encoding glutamate receptors, the proteins of voltage-gated calcium channels, and the D2 dopamine receptors gene; the last one encodes the receptor crucial for the action of antipsychotic agents. Additionally, understanding the interrelations between the glutamatergic pathway dysfunction and dopamine signaling abnormalities may be necessary for comprehending how psychotic symptoms arise [2].

Commonly found single nucleotide variations seem to have lower importance for the development of schizophrenia than rare structural genomic variants. However, they are not highly specific to schizophrenia [44]. Structural disorders that are associated with the occurrence of schizophrenia have been identified, including 11 copy number variants (CNVs), which are not very common, but increase the risk of the disease. It is estimated that CNVs are responsible for about 5% of schizophrenia [2]. The occurrence of some mutations, such as DISC1 translocation, were described only in one family in Scotland [45]. Microdeletion in the 22q11.2 region causes presence of the schizophrenia symptoms in 25–35% of the gene mutation carriers [46].

In the epidemiological studies, attention was also paid to the role of environmental factors predisposing to schizophrenia development [41]. These include, present at the early stage, e.g.,: premature birth, fetal hypoxia, emergency cesarean section, fetal malnutrition, preeclampsia, or infections during pregnancy [47,48]; and these occurring later: growing up in an urban environment; traumatic experiences—mainly when they occur in childhood; or being an immigrant and using psychoactive substances—mostly amphetamines, cocaine, and cannabinoids. Environmental factors alone do not significantly increase the risk of schizophrenia. Additionally, they are not indispensable to developing the disease [48].

It is believed that schizophrenia is a multifactorial disease; genetic and environmental factors are involved in its development, and complex interactions exist between them. As a result, the environmental factor becomes significant with the simultaneous presence of the appropriate genetic variant and vice versa [49], as is in the case of diabetes, atherosclerosis, or other diseases of complex etiology [50]. Some genes involved in brain development and associated with schizophrenia risk are also found in other psychiatric disorders, including neurodevelopmental disorders such as autism [2,40]. The results of these studies are consistent with the widely accepted neurodevelopmental theory of schizophrenia, which was formulated over 30 years ago independently by Robin Murray and Daniel Weinberger [51]; according to it, schizophrenia occurs in genetically predisposed people who, in the prenatal, perinatal, or early life period, had disturbances in brain development under the influence of deleterious factors [52]. The reasons why the symptoms of schizophrenia, unlike other neurodevelopmental diseases, appear much later are unclear. Latency may be caused by a long-term process of the cerebral cortex development, which is still not completed during the manifestation of symptoms [44]. The maturation of the human cortex is little known. However, it is assumed that a significant part of the development is creating a subtle balance between excitatory and inhibitory neurons, which is disturbed in schizophrenia [44,52]. In childhood, the disturbed balance can be compensated [52]. Decompensation may appear later due to the growing modulating effect of dopamine in the cortex in adolescence [44].

### 5.1. Neurotransmission Disorders in Schizophrenia

#### 5.1.1. Dopamine Hypothesis of Psychosis

The linking of dopaminergic transmission disorders with the symptoms of schizophrenia became the basis for the formulation of the dopamine hypothesis of schizophrenia over 50 years ago—the first, but still considered the most significant, attempt to explain the symptoms of schizophrenia [53,54]. This theory, along with many research results, was subject to significant changes and became more complicated [53], but dopamine remained the most important neurotransmitter in schizophrenia. In its original form, the dopamine theory was the result of observing the antipsychotic effects of compounds such as reserpine, which, through its mechanism of action, reduces the level of monoamines—including dopamine—released into the synapse [53]. Conversely, increasing the release of monoamines, including dopamine, after amphetamine administration increased the symptoms of psychosis [53,54]. Dopaminergic hyperactivity was a fairly simple but logical explanation for the pathogenesis of schizophrenia, supported by the clinical effectiveness of D2 receptor-blocking drugs [53]. The effectiveness of these drugs may have seemed even greater, because, at that time, the picture of schizophrenia was dominated by the views of Kurt Schneider, which, according to whom, the psychotic symptoms were the first ranked symptoms of schizophrenia [51]. A huge step in the evolution of the dopamine theory was a paper published in 1991 by Davis et al. [55]. The authors of this review not only narrowed the dopaminergic hyperactivity to the mesolimbic area, but also drew attention to the hypoactivity of dopaminergic neurons in the prefrontal cortex, and, most importantly, linked these phenomena with the symptoms of schizophrenia, positive and negative, respectively. The vast amount of conducted research has allowed for better insight and understanding of the complexity of neurotransmission processes in the brain [53]. Attention was also drawn to the dominant presynaptic nature of disorders in dopaminergic transmission, consisting of the increased synthesis and release of dopamine, rather than to the dysfunction of the postsynaptic D2/D3 receptors themselves [56]. In addition to the classic dopamine theory, other theories have been developed indicating the involvement of other neurotransmitters apart from dopamine, such as glutamate and serotonin, in the development of psychosis. Importantly, however, these three theories complement rather than exclude each other [54].

#### 5.1.2. Glutamate Hypothesis of Psychosis

This theory assumes that one of the causes of excessive dopaminergic activity in the striatum is the hypofunction of the NMDA glutamate receptor located on GABA-ergic interneurons in the prefrontal cortex [54,57]. Glutamate is the major excitatory neurotransmitter found in the brain [58]. NMDA receptors are ligand-gated heterotetrameric ion channels; the activation process is complex. In addition to glutamate, the channel opening requires the attachment of D-serine or L-glycine and the removal of the Mg^2+^ block [57]. The basis for the formulation of the glutamate hypothesis was, as in the case of the dopamine hypothesis, indirect observations indicating the involvement of glutamate in the symptoms of psychosis. Contrary to dopamine, however, psychotic symptoms appear after lowering the effect of glutamate. Phencyclidine (PCP) and ketamine, both non-competitive NMDA receptor antagonists, induce psychotic as well as negative and cognitive symptoms in humans when administered [59]. An association has also been shown between the systematic administration of the NMDA receptor antagonists (ketamine, PCP, MK-801) and increased dopamine release in the striatum (in animal models and humans). Oppositely, in some animal species, long-term phencyclidine administration resulted in a decrease in dopamine release in the prefrontal area. Such differences in dopaminergic transmission in various dopaminergic pathways are considered typical for schizophrenia [57]. The administration of the NMDA receptor antagonists primarily affects the population of these receptors on the GABA-ergic interneurons of the cortex and the hippocampus [57]. GABAergic interneurons play a crucial role in modulating the balance between excitation and inhibition in the cortex. Inhibition of their activity results in the disinhibition of excitatory neurons [60]. Increased activity of the excitatory neurons, through released glutamate, can lead to increased stimulation and overactivity of dopaminergic neurons of the mesolimbic pathway [54]. An interesting piece of evidence favoring glutamate hypofunction on GABA interneurons in schizophrenia may be the symptoms in patients suffering from the anti-NMDA receptor encephalitis. In this disorder, as a result of the action of antibodies on NMDA receptors, their number on the neuron’s surface is reduced. As a result, apart from neurological symptoms such as seizures and movement disorders, most patients had symptoms typical of schizophrenia—bizarre behavior, agitation, anxiety, and psychotic symptoms [61]. The role of the NMDA receptor may also be indicated by changes in the genes encoding the NMDA receptor subunits, revealed in patients with schizophrenia [43,57].

#### 5.1.3. Serotonin Hypothesis of Psychosis

The possible influence of serotonin transmission on the symptoms of schizophrenia was put forward based on the hallucinogenic properties of LSD observed more than half a century ago. It was found that lysergic acid diethylamide (LSD) caused hallucinations similar to those observed in schizophrenia [62]. The induction of psychotic symptoms was then linked to the effect of this compound on the serotonin system [63]. It is now known that the mechanism of action of LSD is more complex than previously thought. LSD has a pleiotropic mechanism of action including partial agonism at 5-HT2A and antagonism at the 5-HT1A receptors, as well as, at higher doses, it stimulates dopamine D2 receptors and Trace Amine Associate receptor 1 [64]. Despite this, it seems that the hallucinogenic effect of LSD is responsible for the activation of serotonin receptors, mainly 5-HT2A [64,65]. The 5-HT1A and 5-HT2C receptors play a modulating role [64]. Blockade of the 5-HT2A receptor by the selective inhibitor ritanserin has alleviated both psychotic and negative symptoms in schizophrenia [63]. The action of many atypical antipsychotics is associated with an even stronger blockade from the 5-HT2A receptors than from the D2 receptors [66]. So far, the only antipsychotic drug whose action is based entirely on the selective blockade of the 5-HT2A receptor is pimavanserin, registered for the treatment of psychotic disorders in the course of Parkinson’s disease [67]. Due to the pathogenesis of Parkinson’s disease, a lack of the D2 blockade is particularly desirable here. It has been shown that an exceptionally high concentration of the 5-HT2A receptors is found in the cerebral cortex. Stimulating these receptors by agonists alters neuronal function, leading to increased glutamate release in the cerebral cortex [65]. Abnormal stimulation is further transmitted to the dopaminergic neurons of the mesolimbic pathway, with the ultimate effect of excessive dopamine release in this region [54].

## 6. Sex Hormones and Schizophrenia

It is known that there are physiological differences in the morphology and activity of different brain regions in men and women. Studies suggest significant differences in terms of the secretion of neurotransmitters, the number of transporters responsible for reuptake, or the number and subtypes of receptors for particular neurotransmitters (e.g., serotonin or dopamine) between the sexes. This may have important implications for the development of some neuropsychiatric diseases, but may also be the basis for the search for treatments based on sex-specific differences [68]. It is also implied that certain sex chromosome-related genes have a direct effect on the formation and maintenance of sex differences in terms of brain volume, cerebral cortex area, cortical folding, the density and amount of grey matter, the size of certain structures in particular brain areas, or even cerebral blood flow [69,70]. Studies indicate that sexual dimorphism also occurs at the level of immunocompetent cells—e.g., microglia—especially early in life. Immune cells in the brain and the signals they transmit play an important role in some diseases associated with disorders of brain development or plasticity—e.g., schizophrenia [71].

There was an increased density of receptors for sex hormones in certain brain structures. Sex-related differences in terms of laterality may influence the different distribution of receptors for sex hormones between cerebral hemispheres. From as early as the fetal period, sex hormones influence brain development and the appearance of sexual dimorphism of the brain in adults [72,73]. In patients with schizophrenia, these physiological differences may be disrupted and greater sex differences may become apparent, particularly in those regions that are rich in receptors for sex hormones [73,74]. Impact of sex hormones on the risk for schizophrenia starts already during fetal life. Various stressors during pregnancy interfere with the normal effect of hormones on brain development, which may have an impact on the development of schizophrenia in male offspring in future [75]. Normal testosterone levels during fetal life are important for the normal development of the male brain, and abnormal testosterone levels can cause neurodevelopmental disorders [73,75,76]. Sex steroids have the ability to influence neuronal differentiation and synaptic connectivity [77].

Estrogens, testosterone, and PRL, but also pregnenolone, allopregnenolone, DHEA, and DHEA-S may play an important role in the pathogenesis of schizophrenia [78,79]. Sex steroids easily penetrate the CNS. Many of these have the ability to influence neuronal excitability. It is implied that abnormal signaling by neuroactive steroids may contribute to the development of schizophrenia and affect the sex differences observed in this disorder [77].

The effect of estrogens is relatively well described. They are considered to be hormones that protect against the development of schizophrenia and influence the severity of observed symptoms [80,81]. Some authors even use the term “estrogen protection hypothesis” to refer to patients with schizophrenia. Women develop schizophrenia at a later age than men. Moreover, the second peak in incidence in women occurs during menopause when estrogen levels drop significantly. In women, there was also variability in schizophrenia symptoms according to the menstrual cycle, with an increase during low-estrogen phases [82]. The mRNA for estrogen receptors, Erα (hypothalamus and amygdala) and Erβ (hippocampus and entorhinal cortex), were identified in many brain areas; although, their role is not fully understood [73,76]. Estrogens alone, however, are considered to be important neuroactive steroids with neuroprotective effects in various psychiatric or neurological diseases. Among other things, the following effects are postulated: anti-apoptotic activity, reduction of beta-amyloid toxicity, protection against oxidative stress, modulation of the immune and inflammatory response, or influence on mitochondrial function [76,80,81,83]. It is significant that estrogens modulate neurotransmitters in the dopaminergic, glutamatergic, and serotonergic systems, or affect the density of receptors for these neurotransmitters [68,81,84]. However, there is a lack of large clinical studies on the effect of estrogen supplementation on the course of schizophrenia. Studies conducted by various researchers indicated the beneficial effects of estrogens in terms of alleviating the positive and negative symptoms in women [85,86]. There are data indicating a beneficial effect of estrogen supplementation in men with schizophrenia; however, estrogen therapy in men is associated with a high risk of feminization and other estrogen-specific adverse effects, e.g., thromboembolic complications [87]. The use of selective estrogen receptor modulators (SERMs) may be more promising, e.g., the use of raloxifene that shows estrogenic effects in the brain (and bones), and anti-estrogenic effects in other organs such as the mammary gland [87,88]. In postmenopausal women, preliminary studies found a beneficial effect of raloxifene in schizophrenia, which involves achieving faster regression of positive symptoms, negative symptoms, or improved cognitive functions [88,89]. However, not all studies suggest a beneficial effect of raloxifene on cognitive functions in patients with schizophrenia [88,90], and some researchers even reported the worsening of the disease in patients with severe schizophrenia when raloxifene was added to standard therapy for schizophrenia in women [91]. In addition to estrogenic effects in the brain, there are data implying that raloxifene increases the levels of sex hormones, including testosterone, in healthy men, although these data are not completely conclusive. Owens et al. found that administration of raloxifene to men with schizophrenia increased serum testosterone levels, and that effect was not related to receptors for androgens [88].

Testosterone is indirectly (via conversion to estrogens) and directly involved in modulating the mesocorticolimbic system. It was found that the ventral tegmental area (VTA) contained receptors for androgens, which means that testosterone had an effect on the density of dopaminergic neurons in this area [92,93]. Both estrogens and testosterone increase the number of neurons containing tyrosine hydroxylase—an enzyme that is important for dopamine synthesis—and they increase the amount of dopamine in the synaptic gap by, among other things, inhibiting its reuptake. Testosterone is also synthesized locally in the brain, as gonadectomy does not reduce testosterone in the mesocorticolimbic system [93,94]. Studies using testosterone antagonists or inhibitors of testosterone conversion to DHT confirm a significant effect of testosterone on dopaminergic transmission in the VTA area [93].

The role of testosterone in the pathogenesis of schizophrenia is much less understood than the effects of estrogen. Many studies reported lower testosterone levels in men with schizophrenia compared to healthy men of the same age, and that was true for those treated with antipsychotics as well as those not yet receiving such medication [95,96]. However, reduced testosterone levels are not always found in a group of men with schizophrenia. According to single studies, men with schizophrenia may have reduced [95,97], normal [98], or elevated testosterone levels [99,100]. A meta-analysis by Misiak et al. revealed that testosterone levels might differ between different groups of men with schizophrenia. There were elevated total testosterone levels in men with exacerbation of schizophrenia, whereas in the group of men with stable multi-episode schizophrenia treated on an outpatient basis those levels were reduced [77]. Abnormal androgen levels may be a factor in the clinical manifestation of the disease. Low testosterone levels inversely correlate with negative symptoms of schizophrenia; however, there was no such correlation for positive symptoms [101]. Serum testosterone levels may affect excitement levels in men with schizophrenia. Sisek-Šprem et al. compared testosterone levels in schizophrenic patients (showing and not showing symptoms of aggression) with the severity of negative symptoms. Non-aggressive men with low testosterone levels had more severe negative symptoms, which was not found in patients with high levels of aggression [102]. It was also reported that reduced testosterone levels might predict cognitive impairment in men [98].

Some studies focused on the effects of the testosterone precursors DHEA and DHEA-S synthesized in the adrenal gland as adjunctive therapy in schizophrenia. Both compounds are now considered to have potential neuroprotective effects and may affect processes related to neuronal survival or neurogenesis. They also have some antioxidant and anti-inflammatory potential [79,103,104]. However, the results of studies on the role of DHEA and DHEA-S in schizophrenia and its treatment were inconclusive [83,84]. Some researchers found elevated DHEA-S levels in patients with schizophrenia who had not yet started treatment; however, results regarding the correlation of elevated DHEA-S levels with negative symptoms of the disease were discrepant [103,105]. Elevated DHEA-S levels in patients with first-episode schizophrenia were also found in a meta-analysis by Misiak et al. [77], which can be explained as a stress response. DHEA exerts an anti-glucocorticoid action in response to stress, which protects hippocampal cells from neurotoxic effects. DHEA supplementation in patients with moderate-to-severe negative symptoms of schizophrenia lead to significant improvement, especially in women. Various mechanisms of action of DHEA are suggested, e.g., an increased release of dopamine and selective enhancement of dopaminergic activity, and sedative action or action on N-methyl–D-aspartate (NMDA) or sigma receptors [106].

## 7. Schizophrenia and Stress

Stress is now considered an integral component of mental illnesses in general, whether as an environmental factor in the initial pathogenesis or in later stages. There is evidence of altered function of the hypothalamic-pituitary-adrenal (HPA) axis in many psychiatric disorders [83].

Schizophrenia may become a cause of chronic stress. The social perception of mental illness is linked to stigma and a sort of exclusion, both in terms of personal and professional life [107,108,109]. Patients are at a high risk of living alone and facing isolation from society during their lifetime [8]. Social exclusion towards people with schizophrenia may be the result of stigmatizing attitudes towards the diagnosis, or a reaction to observed behaviour resulting from the symptoms of the disease [110]. Mental illness makes it difficult to find a job and a fulfilling partnership. People diagnosed with schizophrenia perceive themselves negatively, have low self-esteem, are overly sensitive to criticism, leave the house less often, and begin to avoid new relationships [111]. Consequently, lack of social contact exacerbates or stabilizes the negative symptoms present in patients with schizophrenia and worsens the prognosis [8,112].

### Effects of Stress on the HPG Axis

Stress leads to several biological changes in the body including the secretion of many neurotransmitters and hormones. Mental or physical stressors activate the locus coeruleus in the brainstem and hypothalamus. They have the ability to trigger a stress response. In response to a stressor, the sympathoadrenal system and the HPA axis are activated.

The adrenal glands are made up of two parts: the medulla and the cortex, which differ in terms of origin, structure, and function. The suprarenal medulla originates from the ectoderm, as does the sympathetic nervous system (SNS), while the adrenal cortex is of mesodermal origin. In the adrenal cortex, the following layers are distinguished: zona reticularis, zona fasciculata, and zona glomerulosa. Glucocorticoids are produced in the zona fasciculata, mineralocorticoids in the zona glomerulosa, and androgens in the zona reticularis.

The occurrence of a stress-inducing factor leads to the activation of the SNS. The SNS stimulates the suprarenal medulla to secrete adrenaline, noradrenaline and opioid peptides (stored with them in the secretory vesicles) into the blood, causing, among other things, pupil dilation, accelerated heart rate and breathing, faster glucose metabolism, and reduced sensitivity to pain. A little later, the HPA axis is activated. The hypothalamus releases corticoliberin into the pituitary portal circulation. CRH increases the secretion of adrenocorticotropic hormone (ACTH) from the pituitary gland into the blood. An increase in blood ACTH levels enhances the release of glucocorticosteroids, including cortisol from the adrenal cortex, and to a lesser extent aldosterone and adrenal androgens [113]. Such changes immediately following a stressor are most often considered beneficial because they allow survival under adverse conditions—a fight-or-flight response to maintain homeostasis, an adaptation to changing environmental conditions [114]. Cortisol, on the basis of negative feedback, inhibits the secretion of CRH and ACTH. However, neurohormonal systems can become overactive in situations where stress is chronic. In such cases, the HPA axis remains active, and cortisol continues to be secreted. In acute stress, cortisol is first increased and then decreased. In contrast, cortisol shows no circadian rhythm during chronic stress [115].

Long-term stress can disrupt the HPG axis. Elevated cortisol levels can inhibit the secretion of GnRH, LH, FSH, and testosterone [116]. An increase in CRH reduces the release of gonadotropins [117,118]. Chronic stress can also lead to an increased release of PRL. The adrenal cortex contains receptors for PRL. PRL enhances adrenal androgen production.

## 8. Pharmacotherapy of Schizophrenia

Schizophrenia as a chronic disease that requires long-term pharmacotherapy. The natural course of schizophrenia is marked by the occurrence of relapses and remissions, especially of positive (productive, psychotic) symptoms [2]. Relapses can occur despite treatment; however, available research results indicate that premature termination of treatment significantly increases this risk [119,120]. In a study assessing the long-term risk of relapse of psychotic symptoms in patients treated for first-episode schizophrenia, after 5 years of improvement, discontinuation of pharmacotherapy resulted in an almost 5-fold increase in the risk of both first- and second-relapse (risk ratio 4.89 and 4.57, respectively) [119]. Therefore, antipsychotic treatment should be continued for 1–2 years after the first ever episode. Patients who have had at least one relapse should follow the treatment for 2–5 years. In the event of subsequent relapses, the administration of medication should continue for at least 5 years, and in some cases, even for life [120]. Systematic (continuous) treatment is more beneficial than intermittent therapy [120]. According to the recommendations of the World Federation of Societies of Biological Psychiatry (WFSBP), the drugs of first choice for the treatment of schizophrenia, regardless of the duration and phase of the disease (first-episode schizophrenia vs. relapse), are antipsychotics [120].

Traditionally, two major groups of antipsychotics are distinguished (Table 1). Historically, older drugs are referred to as first-generation antipsychotics (FGAs), or typical antipsychotics. Currently, the World Health Organization (WHO) classifies 52 compounds [121] in this group: e.g.., haloperidol, perphenazine, chlorpromazine, sulpiride, fluphenazine, zuclopenthixol, flupentixol, pimozide, loxapine, thioridazine, trifluoperazine, levomepromazine, molindone, clopenthixol, and perazine [122]. In contrast, newer antipsychotics are second-generation antipsychotics (SGAs), or atypical antipsychotics [123]. This group includes 16 substances registered in Europe and/or the USA [121]; these include: e.g., clozapine, amisulpride, olanzapine, risperidone, paliperidone, quetiapine, aripiprazole, sertindole, ziprasidone, asenapine, lurasidone, and iloperidone [66,124].

Pharmacotherapy for schizophrenia should be started as soon as possible. The choice of drug should be considered on a case-by-case basis, taking into account among others, the type of predominant clinical symptoms, treatment tolerance, treatment costs, patient preferences, or comorbidities. It is assumed that treatment of a first psychotic episode should start with second-generation antipsychotics and their lower doses. Individuals who were previously untreated respond better to treatment, but have a higher risk of both extrapyramidal and metabolic side effects [124]. Clozapine has a special place—on the one hand, it is considered the most effective drug; on the other hand, its use is associated with a rare but serious risk of agranulocytosis (neutropenia). The risk of neutropenia is 2.9%, and severe neutropenia (agranulocytosis) is 0.8% [125]. Another serious side effect of clozapine can be myocarditis. The incidence of this complication ranges from 1 per 10,000 to 1 per 500, with a mortality rate of up to 50%. The use of clozapine also increases the risk of seizures [123]. For this reason, clozapine is not recommended as a drug of first choice [124].

## 9. Hyperprolactinemia

PRL is a polypeptide hormone produced by lactotroph cells of the anterior lobe of the pituitary gland. Its half-life is approximately 50 min [126]. The hypothalamus inhibits the synthesis and release of PRL by secreting dopamine (PIF). Dopamine, after binding to the membrane D2 receptors of lactotroph cells, causes the inhibition of PRL gene transcription and a decrease in PRL production and secretion. Under the influence of dopaminergic stimulation of lactotroph cells, their proliferation is also inhibited [127].

Physiological PRL levels in men are lower compared to women and should generally be 5–20 ng/mL [128]. Excess PRL may result from functional dysregulation of PRL secretion, damage to the hypothalamus, or the presence of a hormonally active pituitary prolactinoma [129].

In men, hyperprolactinemia can lead to reduced testosterone levels. An increase in PRL interferes with GnRH secretion from the hypothalamus, resulting in inhibition of LH and FSH from the pituitary gland. The effect is to reduce testosterone production in the testes [130]. Hyperprolactinemia in men may be asymptomatic or may present with gynecomastia, galactorrhea, erectile dysfunction, decrease in sex drive, oligospermia, infertility, or decreased bone mineral density [97,131].

### 9.1. Hyperprolactinemia in Antipsychotic-Naïve Patients

Data on PRL levels in patients with schizophrenia, but not yet treated with antipsychotics, are inconclusive. Early studies in many cases revealed reduced or normal PRL levels in patients not yet treated for schizophrenia. Despite finding PRL levels within the range considered normal, there were noticeable differences between PRL levels in patients with schizophrenia according to the type of symptoms of the disease (paranoid, schizoaffective, or disorganized) [132,133]. Other authors found increased PRL levels in patients with first-episode schizophrenia [126,134,135,136]. A small meta-analysis published in 2016 [137] also confirmed that patients with schizophrenia and related conditions had higher PRL levels than controls (people without psychiatric disorders). That tendency was present in both the female and male groups, as also reported in other studies [138]. Pituitary enlargement was observed in people at high risk of developing psychoses [139].

### 9.2. Hyperprolactinemia Associated with Antipsychotic Use

It was proven that pharmacotherapy might become a reason for hyperprolactinemia in people with schizophrenia. By blocking overactive dopaminergic neurons in the mesolimbic pathway, antipsychotics lead to the resolution of positive symptoms of schizophrenia. However, drugs cannot selectively affect a single dopaminergic pathway, and they affect all D2 receptors in the brain [140]. The tuberoinfundibular dopaminergic pathway (system) connects the hypothalamus and pituitary gland. The blockade of D2 receptors in the tuberoinfundibular dopaminergic system by antipsychotics abolishes the physiological dopamine inhibition, and may, consequently, lead to hyperprolactinemia [141]. A clear relationship between the blockade of striatal D2 receptors and increased PRL levels was confirmed in the study by Tsuboi et al. [142] in which the majority of participants (72.6%) were men. Tsuboi et al. reported that the occupation of 73% of striatal D2 receptors is the threshold value at which PRL levels rise.

Antipsychotics vary widely in their potential to induce hyperprolactinemia [127,143]. The risk of inducing hyperprolactinemia by various drugs is presented in the Table 2. First-generation drugs are much more likely than second-generation drugs to increase PRL levels. Hyperprolactinemia was observed in 40–90% of patients taking high-potency first-generation antipsychotics (e.g., butyrophenones and phenothiazines) [127,143]. The blockade of the D2 receptor is the main mechanism of action of FGAs, and the antipsychotic effect is proportional to the degree of blockade of this receptor in the brain [66,144].

The drug binding to the receptor forms a drug-receptor complex [145]:

$$\mathrm{Drug}+\mathrm{Receptor}\overset{K_{\mathrm{on}}}{\underset{K_{\mathrm{off}}}{⇄}}\mathrm{Drug}\;-\;\mathrm{Receptor}\;\mathrm{complex}$$

The rate at which a drug binds to a receptor is dependent on drug concentration, receptor density, and association rate constant (K_on_). In contrast, the rate of dissociation of the drug-receptor complex depends on its concentration and dissociation rate constant (K_off_). The clinical concept of potency of antipsychotics is derived from their affinity for the D2 receptor, and is described by the equilibrium dissociation constant (K_d_). The equilibrium dissociation constant is described by the following relationship [145]:

$$K_{d}=\frac{K_{\mathrm{off}}}{K_{\mathrm{on}}}$$

In vitro studies revealed very large differences in terms of D2 receptor affinity between first- and second-generation antipsychotic compounds. The affinity of haloperidol for the D2 receptor was 100 times higher than that of clozapine. Furthermore, it was confirmed that differences, in terms of affinity, were primarily due to differences in the dissociation constant K_off_, not the constant K_on_ [146]. The differences in the dissociation rate constant K_off_ between SGAs and FGAs gave rise to a hypothesis explaining the lower risk of side effects associated with D2 receptor blockade, including hyperprolactinemia, by atypical drugs. Despite similar rates of D2 receptor occupation by haloperidol and clozapine, the rapid dissociation of clozapine from the receptor makes the blockade transient, allowing endogenous dopamine to stimulate the receptor. Haloperidol, due to its low K_off_ constant, remains bound to the D2 receptor much longer (100 times), making it impossible to overcome the blockade by endogenous dopamine [146].

More recent studies on drug-receptor binding kinetics involving near-physiological conditions in models only partially confirm the previous hypothesis [147]. There is a correlation between the rapid dissociation of compounds from the D2 receptor (K_off_) and their low potential to induce hyperprolactinemia. However, the occurrence of extrapyramidal symptoms (EPS) was dependent on the association rate constant K_on_, not on K_off_. As the authors note, under the conditions of a striatal dopamine synapse that is spatially restricted, a compound that has dissociated from a receptor does not diffuse freely, as in in vitro models, but reconnects to the same or another free receptor, blocking dopaminergic transmission. Hence, the drug-receptor rebinding that depends on the K_on_ constant determines the degree of D2 receptor occupation and blockade.

In the case of the pituitary, both dopamine and antipsychotics are delivered to the anterior lobe of the pituitary gland via portal veins by diffusion. The possibility of free diffusion of the antipsychotic drug after dissociation from the receptor means that rebinding is essentially non-existent. Therefore, compounds that have a rapid dissociation from the D2 receptor (e.g., clozapine, quetiapine) allow dopamine to bind to the D2 receptors of lactotroph cells, and thus inhibit PRL secretion [147].

Serotonin is also involved in the regulation of PRL secretion [140]. Administration of 5-hydroxytryptophan (a serotonin precursor) increased plasma PRL levels in humans. In contrast, simultaneous infusion of cyproheptadine (a serotonin receptor antagonist) significantly reduced the PRL response to 5-hydroxytryptophan [148].

The mechanism of action of second-generation antipsychotics is more complex than that of first-generation antipsychotics. These compounds have a lower affinity and bind more loosely to the D2 receptor, making dissociation from the receptor easier and resulting in a relatively weaker dopaminergic blockade [149]. Moreover, some newer compounds belonging to SGAs (e.g., aripiprazole) show partial agonism towards the D2 receptor under conditions of reduced dopaminergic transmission [150].

The antipsychotic effect of second-generation drugs depends on a relatively stronger serotonergic blockade, mainly via the 5-HT2A receptor [149]. However, the blockade of this receptor alone is not sufficient to produce an antipsychotic effect [146]. Some drugs in this group also interact with other serotonin receptor types—5-HT1A, 5-HT2B/2C, 5-HT6, and 5-HT7—which may broaden their spectrum of action to include beneficial effects on cognitive impairment, and also be responsible for neuroprotective effects [151].

Second-generation antipsychotics are less likely to cause hyperprolactinemia than FGAs. In practice, however, there are exceptions to this rule [140]. Some SGAs, more often than others, can cause an increase in PRL levels. These include risperidone, paliperidone and amisulpride [127,152,153]. Paliperidone (9-hydroxyrisperidone) is an active metabolite of risperidone and has similar properties to it [141,154]. After risperidone administration, hyperprolactinemia occurred in 70–100% of participants [143], and in 94% of men [155]. After administration of amisulpride, there was an increase in PRL levels in all participants and a decrease after the discontinuation of the drug [156].

The different properties of risperidone may be due to the high affinity and slow dissociation of this compound from the D2 receptor [141,147,149]. Another reason may be the limited ability of risperidone and paliperidone to penetrate the blood-brain barrier (BBB), as both compounds are substrates for P-glycoprotein that is important in reducing their levels in the CNS [157]. D2 receptors, which are a target for the antipsychotic effect, are located on the inner side of the BBB, while the pituitary gland is anatomically located outside of it. This is why D2 receptors of the anterior lobe of the pituitary gland are exposed to higher levels of the drug [141].

Amisulpride also shows poor penetration of the BBB [141]. Furthermore, unlike SGAs, it has a much stronger affinity for the D2 receptor than the 5-HT2A [151] receptor, and, in this respect, resembles first-generation drugs more than second-generation drugs [122].

In contrast, chlorpromazine is a first-generation antipsychotic drug that, unusually for that group, has a low risk of inducing hyperprolactinemia [158]. This phenomenon can also be explained by the kinetics of binding of this compound to the receptor, which is marked by rapid K_off_. This allows, under conditions found in the pituitary gland, rapid dissociation of the drug from the D2 receptor, thereby allowing dopamine to overcome its blockade and maintain physiological inhibition of PRL secretion [147].

**Table 2:** The risk of inducing hyperprolactinemia by selected first- and second-generation antipsychotics based on [120,127,141,158,159,160].

High	Moderate	Low or None
Fluphenazine (FGA)	Flupentixol (FGA)	Chlorpromazine (FGA)
Haloperidol (FGA)	Molindone (FGA)	Aripiprazole (SGA)
Pimozide (FGA)	Perphenazine (FGA)	Asenapine (SGA)
Sulpiride (FGA)	Thioridazine (FGA)	Clozapine (SGA)
Zuclopenthixol (FGA)	Trifluoperazine (FGA)	Iloperidone (SGA)
Amisulpride (SGA)	Sertindole (SGA)	Lurasidone (SGA)
Paliperidone (SGA)		Olanzapine (SGA)
Risperidone (SGA)		Quetiapine (SGA)
		Ziprasidone (SGA)

## 10. Schizophrenia and Obesity

Obesity is a common health problem for people with schizophrenia. It is twice as prevalent in people with schizophrenia than in those unaffected [161,162]. Sex may be one of the factors that contribute to the prevalence of obesity in this patient group. While body mass index (BMI) values greater than 25 kg/m^2^ are found in a high proportion of people with schizophrenia regardless of sex, values indicative of obesity (BMI ≥ 30 kg/m^2^) are more common in women [163,164]. Weight gain can also be linked to lifestyle. People with schizophrenia are less physically active compared to the general population [165]. As the disease progresses, they gradually withdraw from social life, leave the house less and less, and begin to lead a sedentary lifestyle [166,167]. They have abnormal eating habits marked by a high intake of saturated fat and a low intake of fiber and fruit [168]. Symptoms of schizophrenia, such as lack of motivation, apathy, and cognitive deficits, can further hinder the development of normal health habits [169].

The choice of pharmacological treatment is an important factor that affects BMI values in schizophrenic patients. Therapy with atypical neuroleptics is more conducive to weight gain compared with classical neuroleptics. However, frequent cases of obesity were also observed in women treated with phenothiazines [170]. It is also postulated that greater weight fluctuations occur in young, previously untreated individuals with a normal baseline BMI [171].

The most likely cause of obesity among people treated with neuroleptics is the receptor mechanisms secondary to their antipsychotic effects. An important link in the appetite regulation is the reward system and serotonergic transmission. The blockade of serotonin 5HT2C receptors (olanzapine, clozapine) leads to a disruption of anorectic action of serotonin in the hypothalamus, resulting in a change in food preference to highly processed foods with a high caloric load [172]. Reduced dopaminergic transmission as a result of D2 receptor inhibition, combined with patients’ subjective feelings associated with schizophrenia, such as despondency or the fear of worsening the symptoms of the disease, may result in a search for measures to compensate for the malaise including hyperphagia [173]. Due to their cholinolytic and antihistaminic effects, some neuroleptics (olanzapine, chlorpromazine) may increase feelings of drowsiness, leading to a decrease in physical activity and thus calorie requirements [171]. Moreover, muscarinic receptor inhibition may have an orexigenic effect on the hypothalamus, thereby increasing the feeling of hunger [172]. People treated with neuroleptics also show an increase in body fat, despite elevated leptin levels, which may indicate a disruption of hypothalamic appetite control mechanisms [171].

### HPG Axis Abnormalities in Obese Men

It was reported that there were reduced levels of total testosterone and reduced levels of serum SHBG in obese men [174,175,176]. There was also a reduced testosterone-to-estradiol ratio level in obese men [177,178].

**Figure 3:**
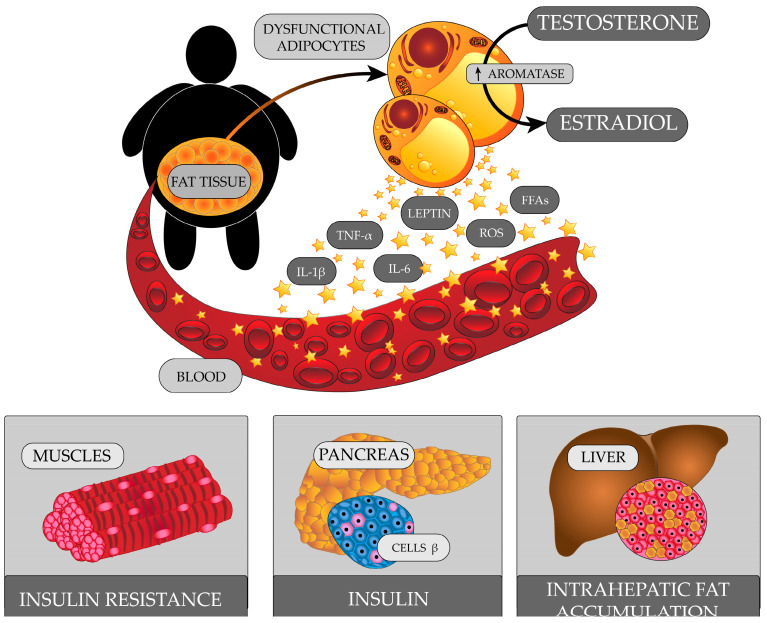
A diagram illustrating the factors involved in the development of HPG axis abnormalities in obese men.

Obese men have an excessive amount of adipose tissue that exhibits metabolic and endocrine activity (Figure 3). In adipocytes, there is an increase in the expression of aromatase, which converts testosterone to estradiol [179]. Increased levels of estradiol affect the HP axis, and, through a negative feedback mechanism, reduce the secretion of GnRH and LH, which consequently leads to insufficient stimulation of Leydig cells to produce testosterone [180].

Adipose tissue releases many biologically active proteins. As adipocyte volume increases, the amount of cytokines and adipokines it produces increases, including tumour necrosis factor α (TNF-α), interleukin 1β (IL-1β), interleukin 6 (IL-6), leptin, resistin, and visfatin. Some of these may affect the HPG axis.

Obese men are found to have increased serum leptin levels [181,182]. Normal leptin levels are important for fertility. They exhibit central and peripheral effects in the reproductive system. Under physiological conditions, leptin stimulates GnRH secretion via kisspeptin [183]. However, in obese people, the hypothalamus becomes resistant to leptin. This leads to reduced expression of the gene for kisspeptin, and consequently, reduced GnRH secretion [184]. It was found that leptin might also modulate the expression and release of gonadotropins [183]. In rat models, there was an inhibitory effect of high leptin levels on testosterone production by Leydig cells with a BMI-dependent effect on pituitary LH secretion [185]. In turn, a decrease in FSH secretion lead to a decrease in synthesis of inhibin B in Sertoli cells. Inhibin B, in the normal state, stimulates Leydig cells to secrete testosterone [186]. Leptin is also involved in regulating testosterone production in the testes. Leydig cells have leptin receptors [187]. As a result of high levels of leptin interacting with its receptor and modulating the activity of the JAK/STAT signaling pathway, the activity of transcription factors that are important for steroidogenic gene expression in Leydig cells is inhibited [188].

Obesity, especially visceral obesity, can cause metabolic syndrome. Although metabolic syndrome is more common in patients treated with antipsychotics, a frequent occurrence of metabolic syndrome is also reported during the first-episode schizophrenia in patients who have not yet received drug treatment [189]. The correlation between schizophrenia and abnormal glucose metabolism was noticed as early as the nineteenth century, when studies found a higher prevalence of diabetes among families with a psychiatric history. Studies in subsequent years reported an increased incidence of impaired glucose tolerance, insulin resistance, and hyperinsulinemia in patients with first-episode schizophrenia [190]. Insulin resistance is a condition of reduced tissue sensitivity to insulin, despite normal or elevated serum levels of this hormone [191]. Insulin is involved in the regulation of the HPG axis centrally and peripherally. Long-term compensatory hyperinsulinemia inhibits the secretion of kisspeptin by hypothalamic neurons, leading to a decrease in GnRH and LH secretion. Sertoli cell function is also inhibited, resulting in reduced inhibin B levels. Insulin resistance is a risk factor for type 2 diabetes (T2D). The occurrence of hyperglycemia further exacerbates oxidative stress and chronic inflammation.

Visceral adipose tissue releases free fatty acids (FFAs) directly into the hepatic circulation [192]. Obese people develop hepatic steatosis [193,194]. The liver is the main site for the production of SHBG. High liver fat content was linked to low SHBG levels [195]. The presence of pro-inflammatory cytokines also reduces SHBG levels [196].

## 11. Schizophrenia and Addictions

Patients with schizophrenia are significantly more likely to abuse psychoactive substances. Substance abuse was reported in this group of people from 3- to up to 9-times-more-often than in the general population [197]. Approximately 50% of people with schizophrenia meet the criteria for addiction at some point in their lives, and thus the criteria for dual diagnosis (DD). Comorbidity in the form of addiction is more common in men [198,199]. Alcohol is one of the most frequently abused substances, both among people with schizophrenia and the general population, because of its easy availability and the fact that it is a legal substance [199,200]. Many hypotheses try to explain the co-occurrence of disorders associated with abusing psychoactive substances, including alcohol, in people with schizophrenia. One of them is the cumulative risk factor hypothesis, which assumes that the abuse of psychoactive substances in people with schizophrenia is due to negative psychological and socio-environmental factors experienced by the patient, such as social exclusion, lack of work opportunities, and often homelessness [200,201]. Another “two-hit” theory explains the genetic susceptibility to the co-occurrence of both disorders [201,202]. The following hypothesis is the “self-medication” theory: people with schizophrenia use psychoactive substances to relieve more intense disease symptoms such as paranoia or auditory hallucinations, or to lessen the side effects of antipsychotics [201,202].

It has been repeatedly demonstrated that liver damages are more prevalent among patients with schizophrenia than in the general population [203,204,205]. Frequent alcohol abuse among patients suffering from mental disorders, including schizophrenia, increases the risk of alcohol-related liver diseases—i.e., alcoholic fatty liver, acute alcoholic hepatitis, or alcoholic cirrhosis. Other possible causes of liver damage include, among others, non-alcoholic fatty liver disease, chronic viral hepatitis, and antipsychotic use [206].

### Effects of Alcohol and Liver Damage on the HPG Axis

Alcohol abuse causes dysfunction in the male reproductive system, affecting each of the three components of the HPG axis. There are several theories explaining the mechanism of the harmful effects of alcohol directly on the male gonads (primary hypogonadism) [207,208], where decreased testosterone production and increased levels of gonadotropins are observed [209].

The direct effect of alcohol on the testes is the main cause of primary hypogonadism in men. In the liver, alcohol is metabolised to acetaldehyde, which in turn is oxidised to acetate, resulting in the production of ROS in the organism [210,211]. The imbalance between oxidation and reduction processes causes oxidative stress, leading to numerous damages at the cellular level, including lipid peroxidation. There are many reports indicating that ROS has a toxic effect on tissues, particularly the liver, but the brain and testes are also damaged [207,211,212,213,214].

The hypothalamus, pituitary gland, and testes produce morphine-like transmitter molecules derived from the opioid group that inhibit the synthesis and/or release of testosterone [215]. An example is the beta-endorphin; its production is increased by excessive alcohol consumption [216], leads to the inhibition of testosterone production and release, and may also enhance apoptosis at the gonadal level causing death of Leydig cells and spermatogenic Sertoli cells [217].

Moreover, ethanol consumed in large amounts inhibits the expression of genes encoding numerous enzymes of the testosterone biosynthetic pathway—i.e., 3β-hydroxysteroid dehydrogenase (3β-HSD) and 17β-hydroxysteroid dehydrogenase (17β-HSD)—as well as the steroidogenic acute regulatory protein (StAR), which is responsible for regulating the transport of cholesterol—a precursor of testosterone—from the outer to the inner mitochondrial membrane [218,219].

Alcohol also adversely affects the HP system causing secondary hypogonadism. Symptoms include low testosterone levels, impaired spermatogenesis, and low gonadotropin levels [209,220]. In this case, alcohol appears to interfere with the GnRH receptor function and/or the interaction of the hormone with its receptor, resulting in reduced LH release. Research reports that acetaldehyde (an intermediate product of alcohol metabolism) interferes with the function of protein kinase C (PKC), which is a key enzyme involved in the production of LH [218,221].

Chronic alcohol abuse by people with schizophrenia can lead to the development of alcoholic cirrhosis. Cirrhosis contributes to hypogonadism and feminisation in men [222]. Such patients experience testicular atrophy, low testosterone levels, decrease in sex drive, impaired spermatogenesis, loss of axillary hair or altered distribution of body hair, and gynecomastia [222,223]. The main cause of testosterone deficiency in men with alcoholic cirrhosis is testicular damage due to the toxic effects of ethanol on Leydig cells. In patients with cirrhosis, hypogonadism is also the result of abnormalities in the HP system. Impairment of LH pulsation and reduction of LH levels are often observed, while the effects of alcohol on GnRH pulsation have not yet been investigated [224]. Furthermore, in cirrhosis, there are often elevated levels of estrogens [225,226]. The increased ethanol-induced aromatase activity—an enzyme present in fat, kidneys, muscles and also in the liver—result in the conversion of testosterone to estradiol and androstendione to estrone [224]. Increased estrogen levels stimulate the production of SHBG—a beta-globulin produced by hepatocytes that binds sex hormones [226,227,228]. An increase in SHBG levels contributes to a decrease in serum free testosterone and dihydroxytestosterone binding, leading to the development of hypogonadism and feminising features in men with cirrhosis [223].

## 12. Conclusions

Despite the enormous progress in knowledge in the field of pathogenesis and the involvement of various neurotransmitters and hormones, including sex hormones, the interaction of genetic and environmental factors, and progress in the pharmacotherapy of schizophrenia, there is still much to be explained. This review tried to explain and highlight recent advances in the field of these problems in men with schizophrenia, focusing on abnormalities of the hypothalamus-pituitary-gonadal axis.

Various endocrine disorders were found in men with schizophrenia. Individual studies often produced divergent results. Reduced, normal, or elevated blood levels of testosterone were described, similarly in the case of PRL, DHEA, DHEA-S, and estradiol. This may be due to the complexity of the processes that affect hormone levels, as well as the number of factors that may play a role in the pathogenesis and onset of schizophrenia symptoms. It is still a matter of dispute which endocrine disorders are the cause, and which are the consequence of the onset of the disease. Further research on this topic is considered imperative to gain deep insights into this difficult topic and allow the personalization of the diagnosis and treatment of schizophrenia.

Successful treatment of schizophrenia is associated with improved prognosis. Antipsychotics are the drugs of first choice in the treatment of schizophrenia. However, their use may be associated with side effects such as gynecomastia, erectile dysfunction, and weight gain. Earlier diagnosis of endocrine disorders and modification of some of the factors that cause them (e.g., changing from one antipsychotic drug to another, reducing body weight, abstaining from alcohol) may improve the cooperation of men with schizophrenia and their continuation of treatment.

## Figures and Tables

**Table 1:** Division of antipsychotics by generation based on [66,122,124].

First-Generation Antipsychotics (FGAs) (Selected)	Second-Generation Antipsychotics (SGAs) (Selected)
Chlorpromazine	Amisulpride
Clopenthixol	Aripiprazole
Flupentixol	Asenapine
Fluphenazine	Clozapine
Haloperidol	Iloperidone
Levomepromazine	Lurasidone
Loxapine	Olanzapine
Molindone	Paliperidone
Perazine	Quetiapine
Perphenazine	Risperidone
Pimozide	Sertindole
Sulpiride	Ziprasidone
Thioridazine	
Trifluoperazine	
Zuclopenthixol	

## Data Availability

Not applicable.
